# Supplementary material for: Market integration reduces kin density in women’s ego-networks in rural Poland
Source: Nat Commun. 2020 Jan 14;11:266. doi: 10.1038/s41467-019-14158-2 (PMC6959218; doi:10.1038/s41467-019-14158-2)
Supplement: Supplementary file 1 — Supplementary Information [file 41467_2019_14158_MOESM1_ESM.pdf]

Supplementary Information

"Market Integration Reduces Kin Density in Women's Ego-Networks in Rural Poland"

Heidi Colleran

Contents:

Supplementary Note 1

Supplementary Tables 1 to 10

Supplementary Note 1

The study was carried out in a sample of communities (21 villages and one town) randomly drawn from four neighbouring municipalities in a southern Polish district in the province of Małopolska, containing a total of 34 potential study populations. All 22 of the sampled communities agreed to participate. I did not have access to a census list of names or a map of households, so within each community every third house was sampled, with every adult woman (≥18 years) present in the house invited to take part in the survey. This strategy was further stratified in the town, by randomly selecting streets from a list obtained from the local government and approaching every third house/apartment on that particular street. I returned to houses that were unoccupied at the time of selection on up to three occasions. All consenting adult women were interviewed. All eligible women who declined to take part were noted as non-responders, as were women ineligible to take part due to age or illness. 52.4% to 89.4% of respondents who were approached in any village agreed to an interview, and the average response rate for the entire sample was 75% (total interviews ÷ [total responders + non-responders]). My sampling strategy means that important variables such as age are approximately normally distributed in all groups. The sample should not be considered representative of the country as a whole, but of the wider rural population of this particular region.

Social Network Questionnaire

The social network questionnaire was administered as part of a broad-ranging semi-structured interviews with women. The questionnaire section for eliciting the network data (questions 30, 31 and 34 of a larger interview) is given below, first in Polish and then in English. Supplementary Table 1 below provides descriptive statistics of the networks.

30. Proszę wymienić do pięcu kobiet, które może nazwać Pani swoimi przyjaciółkami. Kobiet bliskich, z którymi może Pani i lubi rozmawiać o ważnych, osobistych sprawach (na przykład o dzieciach, rodzinie, zdrowiu, albo innych rzeczach). Mogą być z Panią spokrewnione albo pochodzić z poza Pani rodziny. Mogą by z tej miejscowości albo nie. Nie musi wymieniać Pani pięciu osób.

[Jeśli NIE MA]: Dlaczego nie? (zaznacz jedno)

Nie rozmawiam o moich prywatnych sprawach	Nie mam nikogo bliskiego	Nie chce rozmawiać o tym	inny powód:

31.	1	2	3	4	5
a. Wiek					
b. Ile lat się Panie znają?					
c. Czy jest z pani rodziny? (1=tak, 2=nie)	1 2	1 2	1 2	1 2	1 2
d. Pokrewieństwo (kody niżej)					
e. Skąd pochodzi (kody niżej)	1 2 3 4 5	1 2 3 4 5	1 2 3 4 5	1 2 3 4 5	1 2 3 4 5
f. Gdzie obecnie mieszka (kody niżej)	1 2 3 4 5	1 2 3 4 5	1 2 3 4 5	1 2 3 4 5	1 2 3 4 5
g. Czy posiada gospodarstwo rolne? (1=tak, 2=nie)	1 2	1 2	1 2	1 2	1 2
h. Wykształcenie [KARTA 1]					
i. Czy kiedykolwiek pracowała zarobkowo? (1=tak, 2=nie)	1 2	1 2	1 2	1 2	1 2
j. Czy kiedykolwiek mieszkała zagranica? (1=tak, 2=nie)	1 2	1 2	1 2	1 2	1 2
k. Ile ma dzieci?					
l. Wiek najstarszego dziecko					
m. Czy **kiedykolwiek w zyciu** stosowała naturalne metody uniknąć zajścia w ciążę? (1=tak, 2=nie, 3=NW)	1 2 3	1 2 3	1 2 3	1 2 3	1 2 3
n. Czy **kiedykolwiek** w zyciu stosowała nie-naturalne metody uniknąć zajścia w ciążę (np. preserwatywach)? (1=tak, 2=nie,3=NW)	1 2 3	1 2 3	1 2 3	1 2 3	1 2 3
o. Jak często Pani się widzi z ta osoba? (kody niżej)	1 2 3 4 5	1 2 3 4 5	1 2 3 4 5	1 2 3 4 5	1 2 3 4 5
p. Jak często się kontaktujecie? (np telefonicznie)(kody niżej)	1 2 3 4 5	1 2 3 4 5	1 2 3 4 5	1 2 3 4 5	1 2 3 4 5
q. Jak blisko ze sobą jesteście? [KARTA 2]	1 2 3 4	1 2 3 4	1 2 3 4	1 2 3 4	1 2 3 4

KODY DO PYTANIA **D. POKREWIEŃSTWO**
1= SIOSTRA 4= CORKA 7= INNE Z MOIM STRONE
2= BRATOWA 5= TESCIOWA 8= INNE Z STRONE MEZA
3= MATKA 6= KUZINKA 9= SYNOWA

KODY DO PYTANIA **.E & .F MIESJSCE ZAMIESZKANIA**
1= TUTAJ 4= MIASTO pow. 100 tys.
2= INNA WSI 5= INNY KRAJ
3= INNA MIASTO

KODY DO PYTANIA **.O & .P JAK CZESTO SIE …**
1= (Blisko) CODZIENNIE 4= RAZ NA MIESIĄC
2= RAZ NA TYDZIEŃ 5= KILKA RAZY W ROKU
3= KILKA RAZY W MIESIĄCU

34. Proszę wskazać zależności pomiędzy kobietami, o których Pani wcześniej wspomniała. [Wpisz imiona kobiet w pola. Narysuj linie reprezentujące związki pomiędzy poszczególnymi osobami.]

Czarny = Spokrewnieni	Zielony = Rodzina przez malzenstwo	Czerwony = Bliscy przyjaciele	Niebieski = Tylko znajomi

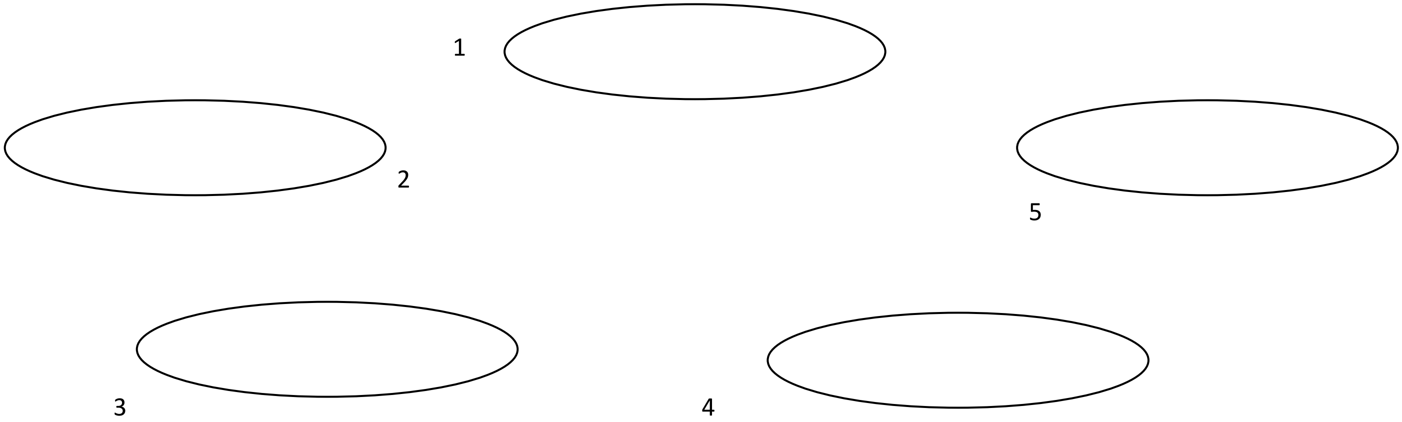

30. Please name up to five women, who you could call your friends. These are women you are close to, and who you can talk to about important personal matters (for example about your children, family, health, or other things). These women can be your relatives or come from outside your family. They can be from this village or anywhere else. You don't have to name five.

[If NONE]: Why not? (mark one)

I don't talk about my private life	I don't have anybody close	I don't want to talk about this	Other reason:

31.	1 (name)	2 (name)	3 (name)	4 (name)	5 (name)
a. Age					
b. How many years have you known each other?					
c. Is she a member of your family? (circle 1=yes, 2=no)	1 2	1 2	1 2	1 2	1 2
d. Relationship (if related) (codes below)					
e. Where is she from? (codes below)	1 2 3 4 5	1 2 3 4 5	1 2 3 4 5	1 2 3 4 5	1 2 3 4 5
f. Where does she currently live? (codes below)	1 2 3 4 5	1 2 3 4 5	1 2 3 4 5	1 2 3 4 5	1 2 3 4 5
g. Does she live on/have a farm? (circle 1=yes, 2=no)	1 2	1 2	1 2	1 2	1 2
h. Education level [KARTA 1]					
i. Did she ever work for wages? (circle 1=yes, 2=no)	1 2	1 2	1 2	1 2	1 2
j. Did she ever live abroad? (circle 1=yes, 2=no)	1 2	1 2	1 2	1 2	1 2
k. How many children does she have?					
l. Age of eldest child					
m. Did she **ever in her life** use natural methods to avoid getting pregnant? (1=yes, 2=no, 3= Don't know)	1 2 3	1 2 3	1 2 3	1 2 3	1 2 3
n. Did she **ever in her life** use other/non-natural methods to avoid getting presents (e.g. condoms, pills)? (1=yes, 2=no,3=Don't know)	1 2 3	1 2 3	1 2 3	1 2 3	1 2 3
o. How often do you see each other? (codes below)	1 2 3 4 5	1 2 3 4 5	1 2 3 4 5	1 2 3 4 5	1 2 3 4 5
p. How often do you contact each other? (e.g by telephone) (codes below)	1 2 3 4 5	1 2 3 4 5	1 2 3 4 5	1 2 3 4 5	1 2 3 4 5
q. How close are you? [SHOWCARD 2]	1 2 3 4	1 2 3 4	1 2 3 4	1 2 3 4	1 2 3 4

CODES FOR Q.D - RELATIONSHIP
1= SISTER 4= DAUGHTER 7= OTHER ON MY SIDE OF THE FAMILY
2= SISTER IN LAW 5= MOTHER IN LAW 8= OTHER ON MY HUSBANDS SIDE OF THE FAMILY
3= MOTHER 6= COUSIN 9= DAUGHTER IN LAW

CODES FOR Q.E & F – PLACE OF RESIDENCE
1= THIS VILLAGE/TOWN 4= TOWN OVER 100,000
2= OTHER VILLAGE 5= OTHER COUNTRY
3= OTHER TOWN

CODES FOR Q.O & P – FREQUENCY OF CONTACT
1= (Almost) EVERY DAY 4= ONCE A MONTH
2= ONCE A WEEK 5= COUPLE OF TIMES A YEAR
3= A FEW TIMES A MONTH

Person ID: Group ID: Family ID:

34. Please help me to connect these women that you have mentioned. [Write friends names in the circles. Draw the following coloured lines between the relevant women to show their connections to each other. If there is no relationship, do not draw a line]

BLACK = Genealogical relative	GREEN = Affinal relative	RED = Close friend	BLUE = acquaintance

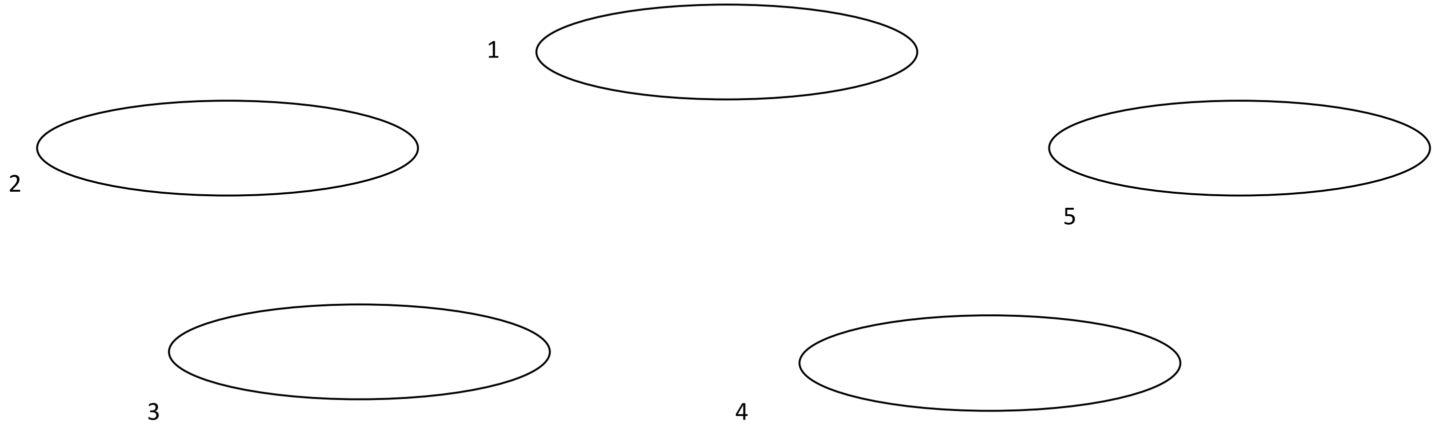

Social network description

Variable	mean	s.d.	range
Mean number of friends (all respondents)	2.92	1.46	0 to 5
Mean number of friends (nominators only)	3.11	1.30	1 to 5
Mean friendship duration (years)	23.18	13.57	0.67 to 78
Mean number of kin	1.37	1.33	0 to 5
Mean number non-kin	1.55	1.32	0 to 5
Relationship to ego	% all alters	% networks	
Mother	3.46%	10.71%	
Sister	17.22%	37.74%	
Daughter	4.99%	9.17%	
Cousin	4.32%	10.82%	
Other genealogical kin	3.86%	9.43%	
Mother-in-law	0.52%	1.49%	
Sister-in-law	7.63%	18.18%	
Daughter-in-law	1.96%	8.16%	
Other affine	3.29%	8.16%	
Origin of friends			
From same village	49.47%		
From other village	36.39%		
From other town	10.23%		
From other city	3.72%		
From another country	0.19%		
Current location of friends			
Resident in same village	52.70%		
Resident in other village	28.54%		
Resident in other town	11.48%		
Resident in other city	5.67%		
Resident in another country	1.62%		
Frequency of face-to-face contact			
Daily	43.69%		
Weekly	25.12%		
Few times a month	14.44%		
Once a month	8.59%		
Few times a year or less	8.16%		
Frequency of phone/email contact			
Daily	66.81%		
Weekly	22.65%		
Few times a month	6.69%		
Once a month	2.82%		
Few times a year or less	1.02%		

Supplementary Table 1. Breakdown of social network characteristics.

	(a) Social Network Size		
**Fixed Effects**	**Estimate**	**Std. Error**	**t value**
Intercept	2.79	0.16	17.80
Relative Age	-0.11	0.06	-1.83
Relative Age sq	-0.05	0.04	-1.46
Relative SES	0.12	0.05	2.32
Relative Material Wealth	0.12	0.04	2.91
Relative Market Integration	0.02	0.04	0.45
Relative Farming Wealth	0.00	0.04	0.03
Farmer Status	0.05	0.08	0.56
Migrant Status	-0.04	0.07	-0.61
Ever Married	0.14	0.11	1.29
Number of Sisters	0.01	0.02	0.45
Average Market Integration	0.04	0.09	0.47
**Random Effects**	**Variance**	**Std.Dev.**	
(Intercept)	0.13	0.36	
Residual	1.98	1.41	
**Model type**	**Linear**		
Number of observations	1972		
Number of groups	22		

	(b) Density						
**Fixed Effects**	**Estimate**	**Std. Error**	**z-value**	**Pr(>|z|)**	**OR**	**95% CI**	
Intercept	0.57	0.14	4.04	0.000	1.77	1.34	2.32
Relative Age	0.20	0.05	3.70	0.000	1.22	1.10	1.36
Relative Age sq	0.34	0.05	7.23	0.000	1.40	1.28	1.53
Relative SES	-0.06	0.05	-1.26	0.209	0.94	0.86	1.03
Relative Material Wealth	-0.07	0.04	-1.88	0.060	0.93	0.86	1.00
Relative Market Integration	0.05	0.03	1.49	0.137	1.05	0.98	1.12
Relative Farming Wealth	0.03	0.04	0.80	0.426	1.03	0.96	1.11
Farmer Status	0.18	0.07	2.54	0.011	1.20	1.04	1.38
Migrant Status	-0.41	0.06	-6.45	0.000	0.67	0.59	0.75
Ever Married	0.49	0.10	4.89	0.000	1.63	1.34	1.99
Number of Sisters	0.09	0.02	4.40	0.000	1.09	1.05	1.13
Average Market Integration	-0.16	0.07	-2.25	0.025	0.85	0.74	0.98
**Random Effects**	**Variance**	**Std.Dev.**					
(Intercept)	0.07	0.27					
Residual	n/a	n/a					
**Model type**	**Binomial**						
Number of observations	1637						
Number of groups	22						

	(c) Kinship Density						
**Fixed Effects**	**Estimate**	**Std. Error**	**z-value**	**Pr(>|z|)**	**OR**	**95% CI**	
Intercept	-1.42	0.10	-14.23	0.000	0.24	0.20	0.29
Relative Age	-0.08	0.04	-2.03	0.042	0.93	0.86	1.00
Relative Age sq	-0.02	0.03	-0.60	0.551	0.98	0.94	1.04
Relative SES	-0.11	0.03	-3.22	0.001	0.90	0.84	0.96
Relative Material Wealth	-0.02	0.03	-0.66	0.509	0.98	0.94	1.03
Relative Market Integration	-0.11	0.02	-4.71	0.000	0.89	0.85	0.94
Relative Farming Wealth	-0.02	0.02	-0.87	0.383	0.98	0.94	1.03
Farmer Status	0.05	0.05	0.99	0.323	1.05	0.95	1.16
Migrant Status	0.02	0.04	0.43	0.666	1.02	0.94	1.11
Ever Married	0.84	0.08	11.03	0.000	2.32	2.00	2.69
Number of Sisters	0.11	0.01	9.04	0.000	1.12	1.09	1.15
Average Market Integration	-0.14	0.05	-2.92	0.004	0.87	0.79	0.95
**Random Effects**	**Variance**	**Std.Dev.**					
(Intercept)	0.03	0.18					
Residual	n/a	n/a					
**Model type**	**Binomial**						
Number of observations	1854						
Number of groups	22						

Supplementary Table 2. Multilevel regression estimates, standard errors and (where appropriate) odds ratios and 95% confidence intervals from the models of (a) Social Network Size, (b) Density and (c) Kinship Density using the full datasets.

(a) Social Network Size

Fixed Effects	Estimate	Std. Error	t value
Intercept	2.97	0.13	22.34
Relative Age	-0.03	0.07	-0.51
Relative Age sq	-0.13	0.04	-2.95
Relative SES	0.12	0.06	2.07
Relative Material Wealth	0.10	0.04	2.27
Relative Market Integration	0.00	0.04	0.04
Relative Farming Wealth	-0.03	0.04	-0.77
Farmer Status	0.05	0.09	0.57
Migrant Status	-0.06	0.07	-0.84
Number of Sisters	0.02	0.02	0.83
Average Market Integration	0.04	0.10	0.40
**Random Effects**	**Variance**	**Std.Dev.**	
(Intercept)	0.17	0.42	
Residual	1.99	1.41	
**Model type**	**Linear**		
Number of observations	1598		
Number of groups	22		

(b) Density

Fixed Effects	Estimate	Std. Error	z-value	Pr(>|z|)	OR	95% CI	
Intercept	1.05	0.12	8.73	0.000	2.86	2.26	3.62
Relative Age	0.22	0.06	3.67	0.000	1.24	1.11	1.40
Relative Age sq	0.27	0.05	5.03	0.000	1.31	1.18	1.45
Relative SES	-0.06	0.05	-1.21	0.225	0.94	0.85	1.04
Relative Material Wealth	-0.10	0.04	-2.16	0.031	0.91	0.83	0.99
Relative Market Integration	0.02	0.04	0.53	0.593	1.02	0.95	1.10
Relative Farming Wealth	0.04	0.04	0.96	0.336	1.04	0.96	1.13
Farmer Status	0.30	0.08	3.66	0.000	1.35	1.15	1.59
Migrant Status	-0.46	0.07	-6.88	0.000	0.63	0.56	0.72
Number of Sisters	0.10	0.02	4.71	0.000	1.11	1.06	1.16
Average Market Integration	-0.17	0.09	-1.90	0.057	0.84	0.70	1.01
**Random Effects**	**Variance**	**Std.Dev.**					
(Intercept)	0.12	0.35					
Residual	n/a	n/a					
**Model type**	**Binomial**						
Number of observations	1315						
Number of groups	22						

(c) Kinship Density

Fixed Effects	Estimate	Std. Error	z-value	Pr(>|z|)	OR	95% CI	
Intercept	-0.67	0.07	-9.56	0.000	0.51	0.45	0.59
Relative Age	-0.14	0.04	-3.63	0.000	0.87	0.80	0.94
Relative Age sq	0.05	0.03	1.76	0.078	1.05	0.99	1.11
Relative SES	-0.12	0.03	-3.54	0.000	0.89	0.83	0.95
Relative Material Wealth	-0.02	0.03	-0.74	0.461	0.98	0.93	1.03
Relative Market Integration	-0.10	0.02	-3.84	0.000	0.91	0.87	0.95
Relative Farming Wealth	-0.03	0.02	-1.13	0.261	0.97	0.93	1.02
Farmer Status	0.06	0.05	1.13	0.257	1.06	0.96	1.18
Migrant Status	0.03	0.04	0.78	0.437	1.03	0.95	1.13
Number of Sisters	0.12	0.01	9.39	0.000	1.13	1.10	1.16
Average Market Integration	-0.13	0.05	-2.84	0.005	0.87	0.80	0.96
**Random Effects**	**Variance**	**Std.Dev.**					
(Intercept)	0.03	0.17					
Residual	n/a	n/a					
**Model type**	**Binomial**						
Number of observations	1497						
Number of groups	22						

Supplementary Table 3. Multilevel regression estimates, standard errors and (where appropriate) odds ratios and 95% confidence intervals from the models of (a) Social Network Size, (b) Density and (c) Kinship Density using the subset of all married women (total n = 1,598).

	(a) Social Network Size		
**Fixed Effects**	**Estimate**	**Std. Error**	**t value**
Intercept	2.86	0.18	16.12
Relative Age	-0.12	0.08	-1.57
Relative Age sq	-0.08	0.05	-1.69
Relative SES	0.13	0.07	1.84
Relative Material Wealth	0.11	0.05	2.15
Relative Market Integration	0.00	0.05	0.07
Relative Farming Wealth	0.00	0.04	0.08
Migrant Status	-0.10	0.09	-1.12
Ever Married	0.19	0.15	1.28
Number of Sisters	0.01	0.03	0.25
Average Market Integration	0.07	0.10	0.70
**Random Effects**	**Variance**	**Std.Dev.**	
(Intercept)	0.15	0.39	
Residual	2.03	1.42	
**Model type**	**Linear**		
Number of observations	1239		
Number of groups	22		

	(b) Density						
**Fixed Effects**	**Estimate**	**Std. Error**	**z-value**	**Pr(>|z|)**	**OR**	**95% CI**	
Intercept	0.48	0.17	2.87	0.004	1.61	1.16	2.23
Relative Age	0.24	0.08	3.18	0.001	1.27	1.10	1.48
Relative Age sq	0.42	0.06	6.94	0.000	1.52	1.35	1.71
Relative SES	-0.02	0.07	-0.26	0.797	0.98	0.86	1.12
Relative Material Wealth	-0.04	0.05	-0.87	0.383	0.96	0.87	1.06
Relative Market Integration	0.03	0.05	0.58	0.561	1.03	0.94	1.13
Relative Farming Wealth	0.03	0.04	0.84	0.403	1.03	0.96	1.12
Migrant Status	-0.47	0.09	-5.47	0.000	0.62	0.52	0.74
Ever Married	0.83	0.14	6.13	0.000	2.30	1.76	3.00
Number of Sisters	0.07	0.02	2.89	0.004	1.07	1.02	1.13
Average Market Integration	-0.15	0.10	-1.59	0.111	0.86	0.71	1.04
**Random Effects**	**Variance**	**Std.Dev.**					
(Intercept)	0.13	0.36					
Residual	n/a	n/a					
**Model type**	**Binomial**						
Number of observations	1015						
Number of groups	22						

	(c) Kinship Density						
**Fixed Effects**	**Estimate**	**Std. Error**	**z-value**	**Pr(>|z|)**	**OR**	**95% CI**	
Intercept	-1.34	0.11	-12.14	0.000	0.26	0.21	0.32
Relative Age	-0.06	0.05	-1.32	0.185	0.94	0.85	1.03
Relative Age sq	-0.01	0.03	-0.24	0.813	0.99	0.94	1.05
Relative SES	-0.12	0.04	-2.73	0.006	0.89	0.82	0.97
Relative Material Wealth	-0.03	0.03	-1.14	0.254	0.97	0.91	1.03
Relative Market Integration	-0.11	0.03	-3.75	0.000	0.89	0.84	0.95
Relative Farming Wealth	-0.03	0.02	-1.18	0.240	0.97	0.93	1.02
Migrant Status	0.13	0.05	2.49	0.013	1.14	1.03	1.27
Ever Married	0.76	0.10	7.73	0.000	2.14	1.76	2.59
Number of Sisters	0.10	0.01	7.00	0.000	1.11	1.08	1.14
Average Market Integration	-0.18	0.05	-3.63	0.000	0.83	0.76	0.92
**Random Effects**	**Variance**	**Std.Dev.**					
(Intercept)	0.03	0.17					
Residual	n/a	n/a					
**Model type**	**Binomial**						
Number of observations	1155						
Number of groups	22						

Supplementary Table 4. Multilevel regression estimates, standard errors and (where appropriate) odds ratios and 95% confidence intervals from the models of (a) Social Network Size, (b) Density and (c) Kinship Density using the subset of all farmers (total n = 1,239).

(a) Social Network Size

Fixed Effects	Estimate	Std. Error	t value
Intercept	2.76	0.17	15.79
Relative Age	-0.11	0.08	-1.50
Relative Age sq	-0.01	0.05	-0.21
Relative SES	0.13	0.07	1.84
Relative Material Wealth	0.15	0.05	3.06
Relative Market Integration	-0.03	0.05	-0.58
Relative Farming Wealth	0.00	0.05	0.06
Farmer Status	0.05	0.10	0.44
Ever Married	0.20	0.12	1.65
Number of Sisters	0.00	0.03	-0.11
Average Market Integration	0.03	0.09	0.33
**Random Effects**	**Variance**	**Std.Dev.**	
(Intercept)	0.10	0.32	
Residual	1.93	1.39	
**Model type**	**Linear**		
Number of observations	1221		
Number of groups	22		

(b) Density

Fixed Effects	Estimate	Std. Error	z-value	Pr(>|z|)	OR	95% CI	
Intercept	0.20	0.17	1.17	0.243	1.22	0.87	1.71
Relative Age	0.24	0.08	2.96	0.003	1.27	1.08	1.49
Relative Age sq	0.49	0.06	7.96	0.000	1.64	1.45	1.85
Relative SES	-0.13	0.07	-1.77	0.077	0.88	0.76	1.01
Relative Material Wealth	0.07	0.05	1.32	0.187	1.07	0.97	1.19
Relative Market Integration	-0.05	0.05	-1.14	0.255	0.95	0.86	1.04
Relative Farming Wealth	-0.19	0.05	-3.69	0.000	0.83	0.75	0.92
Farmer Status	0.37	0.10	3.80	0.000	1.45	1.20	1.76
Ever Married	0.57	0.12	4.90	0.000	1.77	1.41	2.22
Number of Sisters	0.11	0.03	3.92	0.000	1.11	1.06	1.18
Average Market Integration	-0.10	0.08	-1.19	0.234	0.91	0.78	1.06
**Random Effects**	**Variance**	**Std.Dev.**					
(Intercept)	0.07	0.27					
Residual	n/a	n/a					
**Model type**	**Binomial**						
Number of observations	1028						
Number of groups	22						

(c) Kinship Density

Fixed Effects	Estimate	Std. Error	z-value	Pr(>|z|)	OR	95% CI	
Intercept	-1.19	0.12	-9.75	0.000	0.30	0.24	0.39
Relative Age	-0.07	0.05	-1.40	0.162	0.93	0.84	1.03
Relative Age sq	-0.03	0.03	-0.93	0.354	0.97	0.92	1.03
Relative SES	-0.09	0.05	-1.84	0.066	0.92	0.83	1.01
Relative Material Wealth	0.00	0.03	0.15	0.883	1.00	0.94	1.07
Relative Market Integration	-0.16	0.03	-5.12	0.000	0.85	0.80	0.91
Relative Farming Wealth	0.05	0.03	1.66	0.097	1.05	0.99	1.12
Farmer Status	-0.17	0.07	-2.62	0.009	0.84	0.74	0.96
Ever Married	0.83	0.09	9.64	0.000	2.30	1.94	2.72
Number of Sisters	0.09	0.02	5.56	0.000	1.10	1.06	1.13
Average Market Integration	-0.15	0.06	-2.26	0.024	0.86	0.76	0.98
**Random Effects**	**Variance**	**Std.Dev.**					
(Intercept)	0.06	0.24					
Residual	n/a	n/a					
**Model type**	**Binomial**						
Number of observations	1154						
Number of groups	22						

Supplementary Table 5. Multilevel regression estimates, standard errors and (where appropriate) odds ratios and 95% confidence intervals from the models of (a) Social Network Size, (b) Density and (c) Kinship Density using the subset of all non-migrants (total n = 1,221).

	(a) Social Network Size			(b) Density							(c) Kinship Density						
**Fixed Effects**	**Estimate**	**Std. Error**	**t value**	**Estimate**	**Std. Error**	**z-value**	**Pr(>|z|)**	**OR**	**95% CI**		**Estimate**	**Std. Error**	**z-value**	**Pr(>|z|)**	**OR**	**95% CI**	
Intercept	2.74	0.20	13.47	0.70	0.20	3.57	0.000	2.01	1.37	2.94	-1.32	0.14	-9.18	0.000	0.27	0.20	0.35
Relative Age	-0.19	0.08	-2.23	0.24	0.08	3.02	0.003	1.27	1.09	1.48	0.01	0.06	0.21	0.835	1.01	0.91	1.13
Relative Age sq	-0.04	0.06	-0.73	0.41	0.07	5.63	0.000	1.50	1.30	1.73	0.03	0.04	0.64	0.526	1.03	0.95	1.11
Relative SES	0.07	0.07	0.90	-0.11	0.07	-1.62	0.105	0.90	0.79	1.02	-0.10	0.05	-1.95	0.052	0.91	0.83	1.00
Relative Material Wealth	0.09	0.06	1.60	-0.11	0.06	-1.84	0.065	0.90	0.80	1.01	0.05	0.04	1.26	0.208	1.05	0.97	1.13
Relative Market Integration	0.00	0.05	0.09	0.10	0.05	2.12	0.034	1.11	1.01	1.22	-0.12	0.04	-3.45	0.001	0.88	0.83	0.95
Relative Farming Wealth	-0.02	0.06	-0.31	0.10	0.06	1.65	0.100	1.10	0.98	1.23	-0.03	0.04	-0.70	0.484	0.97	0.90	1.05
Farmer Status	0.15	0.11	1.32	0.24	0.10	2.30	0.021	1.27	1.04	1.55	0.08	0.08	1.07	0.284	1.09	0.93	1.26
Migrant Status	-0.09	0.10	-0.95	-0.30	0.09	-3.33	0.001	0.74	0.63	0.89	-0.02	0.06	-0.31	0.755	0.98	0.86	1.11
Ever Married	0.16	0.15	1.08	0.30	0.14	2.15	0.032	1.35	1.03	1.79	0.72	0.11	6.52	0.000	2.05	1.65	2.55
Average Market Integration	0.12	0.11	1.11	-0.11	0.09	-1.27	0.206	0.89	0.75	1.06	-0.16	0.07	-2.46	0.014	0.85	0.75	0.97
**Random Effects**	**Variance**	**Std.Dev.**		**Variance**	**Std.Dev.**						**Variance**	**Std.Dev.**					
(Intercept)	0.16	0.40		0.10	0.31						0.06	0.24					
Residual	1.78	1.34		n/a	n/a						n/a	n/a					
**Model type**	**Linear**			**Binomial**							**Binomial**						
Number of observations	976			825							933						
Number of groups	22			22							22						

Supplementary Table 6. Multilevel regression estimates, standard errors and (where appropriate) odds ratios and 95% confidence intervals from the models of (a) Social Network Size, (b) Density and (c) Kinship Density using the subset of all women with one or zero sisters (total n = 976).

	(a) Social Network Size		
**Fixed Effects**	**Estimate**	**Std. Error**	**t value**
Intercept	2.77	0.18	15.51
Relative Age	-0.11	0.06	-1.84
Relative Age sq	-0.05	0.04	-1.45
Relative SES	0.12	0.05	2.31
Relative Material Wealth	0.12	0.04	2.91
Relative Market Integration	0.02	0.04	0.45
Relative Farming Wealth	0.00	0.04	0.04
Farmer Status	0.04	0.08	0.53
Migrant Status	-0.04	0.07	-0.61
Ever Married	0.14	0.11	1.29
Number of Sisters	0.01	0.02	0.44
Population Density	0.13	0.43	0.30
**Random Effects**	**Variance**	**Std.Dev.**	
(Intercept)	0.14	0.37	
Residual	1.98	1.41	
**Model type**	**Linear**		
Number of observations	1972		
Number of groups	22		

	(b) Density						
**Fixed Effects**	**Estimate**	**Std. Error**	**z-value**	**Pr(>|z|)**	**OR**	**95% CI**	
Intercept	0.65	0.16	4.05	0.000	1.91	1.40	2.62
Relative Age	0.20	0.05	3.75	0.000	1.22	1.10	1.36
Relative Age sq	0.34	0.05	7.24	0.000	1.40	1.28	1.54
Relative SES	-0.06	0.05	-1.22	0.222	0.95	0.86	1.03
Relative Material Wealth	-0.07	0.04	-1.86	0.063	0.93	0.86	1.00
Relative Market Integration	0.05	0.03	1.51	0.131	1.05	0.98	1.13
Relative Farming Wealth	0.03	0.04	0.80	0.421	1.03	0.96	1.11
Farmer Status	0.19	0.07	2.61	0.009	1.21	1.05	1.39
Migrant Status	-0.40	0.06	-6.43	0.000	0.67	0.59	0.76
Ever Married	0.49	0.10	4.90	0.000	1.64	1.34	1.99
Number of Sisters	0.09	0.02	4.40	0.000	1.09	1.05	1.13
Population Density	-0.45	0.35	-1.30	0.194	0.64	0.32	1.26
**Random Effects**	**Variance**	**Std.Dev.**					
(Intercept)	0.09	0.30					
Residual	n/a	n/a					
**Model type**	**Binomial**						
Number of observations	1637						
Number of groups	22						

	(c) Kinship Density						
**Fixed Effects**	**Estimate**	**Std. Error**	**z-value**	**Pr(>|z|)**	**OR**	**95% CI**	
Intercept	-1.30	0.11	-11.52	0.000	0.27	0.22	0.34
Relative Age	-0.07	0.04	-1.98	0.048	0.93	0.86	1.00
Relative Age sq	-0.02	0.03	-0.63	0.530	0.98	0.94	1.03
Relative SES	-0.10	0.03	-3.19	0.001	0.90	0.84	0.96
Relative Material Wealth	-0.02	0.03	-0.66	0.513	0.98	0.94	1.03
Relative Market Integration	-0.11	0.02	-4.70	0.000	0.89	0.85	0.94
Relative Farming Wealth	-0.02	0.02	-0.84	0.402	0.98	0.94	1.03
Farmer Status	0.05	0.05	0.91	0.364	1.05	0.95	1.16
Migrant Status	0.02	0.04	0.47	0.637	1.02	0.94	1.11
Ever Married	0.84	0.08	11.00	0.000	2.31	1.99	2.69
Number of Sisters	0.11	0.01	9.07	0.000	1.12	1.09	1.15
Population Density	-0.60	0.24	-2.49	0.013	0.55	0.34	0.88
**Random Effects**	**Variance**	**Std.Dev.**					
(Intercept)	0.04	0.20					
Residual	n/a	n/a					
**Model type**	**Binomial**						
Number of observations	1854						
Number of groups	22						

Supplementary Table 7. Multilevel regression estimates, standard errors and (where appropriate) odds ratios and 95% confidence intervals from the models of (a) Social Network Size, (b) Density and (c) Kinship Density using population density instead of market integration as a community level predictor in the models.

	(a) Social Network Size		
**Fixed Effects**	**Estimate**	**Std. Error**	**t value**
Intercept	3.57	0.70	5.13
Relative Age	-0.11	0.06	-1.84
Relative Age sq	-0.05	0.04	-1.45
Relative SES	0.12	0.05	2.33
Relative Material Wealth	0.12	0.04	2.91
Relative Market Integration	0.02	0.04	0.45
Relative Farming Wealth	0.00	0.04	0.01
Farmer Status	0.05	0.08	0.60
Migrant Status	-0.04	0.07	-0.62
Ever Married	0.15	0.11	1.32
Number of Sisters	0.01	0.02	0.50
Average Market Integration	0.01	0.09	0.08
Average Completed Fertility	-0.20	0.18	-1.14
**Random Effects**	**Variance**	**Std.Dev.**	
(Intercept)	0.13	0.36	
Residual	1.98	1.41	
**Model type**	**Linear**		
Number of observations	1972		
Number of groups	22		

	(b) Density						
**Fixed Effects**	**Estimate**	**Std. Error**	**z-value**	**Pr(>|z|)**	**OR**	**95% CI**	
Intercept	0.12	0.53	0.22	0.825	1.12	0.40	3.19
Relative Age	0.20	0.05	3.71	0.000	1.22	1.10	1.36
Relative Age sq	0.34	0.05	7.23	0.000	1.40	1.28	1.53
Relative SES	-0.06	0.05	-1.26	0.207	0.94	0.86	1.03
Relative Material Wealth	-0.07	0.04	-1.88	0.060	0.93	0.86	1.00
Relative Market Integration	0.05	0.03	1.48	0.139	1.05	0.98	1.12
Relative Farming Wealth	0.03	0.04	0.82	0.411	1.03	0.96	1.11
Farmer Status	0.18	0.07	2.51	0.012	1.20	1.04	1.38
Migrant Status	-0.41	0.06	-6.45	0.000	0.67	0.59	0.75
Ever Married	0.49	0.10	4.87	0.000	1.63	1.34	1.99
Number of Sisters	0.09	0.02	4.36	0.000	1.09	1.05	1.13
Average Market Integration	-0.14	0.07	-1.89	0.059	0.87	0.75	1.01
Average Completed Fertility	0.12	0.13	0.88	0.380	1.13	0.86	1.47
**Random Effects**	**Variance**	**Std.Dev.**					
(Intercept)	0.07	0.26					
Residual	n/a	n/a					
**Model type**	**Binomial**						
Number of observations	1637						
Number of groups	22						

	(c) Kinship Density						
**Fixed Effects**	**Estimate**	**Std. Error**	**z-value**	**Pr(>|z|)**	**OR**	**95% CI**	
Intercept	-1.73	0.36	-4.77	0.000	0.18	0.09	0.36
Relative Age	-0.07	0.04	-2.02	0.044	0.93	0.86	1.00
Relative Age sq	-0.02	0.03	-0.60	0.548	0.98	0.94	1.04
Relative SES	-0.11	0.03	-3.22	0.001	0.90	0.84	0.96
Relative Material Wealth	-0.02	0.03	-0.65	0.517	0.98	0.94	1.03
Relative Market Integration	-0.11	0.02	-4.72	0.000	0.89	0.85	0.94
Relative Farming Wealth	-0.02	0.02	-0.85	0.396	0.98	0.94	1.03
Farmer Status	0.05	0.05	0.96	0.335	1.05	0.95	1.16
Migrant Status	0.02	0.04	0.43	0.664	1.02	0.94	1.11
Ever Married	0.84	0.08	11.02	0.000	2.32	1.99	2.69
Number of Sisters	0.11	0.01	8.98	0.000	1.12	1.09	1.15
Average Market Integration	-0.13	0.05	-2.53	0.012	0.88	0.80	0.97
Average Completed Fertility	0.08	0.09	0.88	0.381	1.08	0.91	1.30
**Random Effects**	**Variance**	**Std.Dev.**					
(Intercept)	0.03	0.18					
Residual	n/a	n/a					
**Model type**	**Binomial**						
Number of observations	1854						
Number of groups	22						

Supplementary Table 8. Multilevel regression estimates, standard errors and (where appropriate) odds ratios and 95% confidence intervals from the models of (a) Social Network Size, (b) Density and (c) Kinship Density including community average completed fertility as a covariate at the community level of the models.

	(a) Social Network Size			(b) Density							(c) Kinship Density						
**Fixed Effects**	**Estimate**	**Std. Error**	**t value**	**Estimate**	**Std. Error**	**z-value**	**Pr(>|z|)**	**OR**	**95% CI**		**Estimate**	**Std. Error**	**z-value**	**Pr(>|z|)**	**OR**	**95% CI**	
Intercept	3.50	0.48	7.23	1.04	0.37	2.82	0.005	2.82	1.37	5.79	-1.39	0.28	-5.02	0.000	0.25	0.14	0.43
Relative Age	-0.11	0.06	-1.81	0.20	0.05	3.70	0.000	1.22	1.10	1.36	-0.08	0.04	-2.03	0.042	0.93	0.86	1.00
Relative Age sq	-0.05	0.04	-1.45	0.34	0.05	7.23	0.000	1.40	1.28	1.53	-0.02	0.03	-0.60	0.551	0.99	0.94	1.04
Relative SES	0.12	0.05	2.34	-0.06	0.05	-1.27	0.206	0.94	0.86	1.03	-0.11	0.03	-3.21	0.001	0.90	0.84	0.96
Relative Material Wealth	0.12	0.04	2.92	-0.08	0.04	-1.90	0.057	0.93	0.86	1.00	-0.02	0.03	-0.66	0.509	0.98	0.94	1.03
Relative Market Integration	0.02	0.04	0.45	0.05	0.03	1.50	0.134	1.05	0.98	1.13	-0.11	0.02	-4.71	0.000	0.89	0.85	0.94
Relative Farming Wealth	0.00	0.04	0.02	0.03	0.04	0.79	0.428	1.03	0.96	1.11	-0.02	0.02	-0.87	0.383	0.98	0.94	1.03
Farmer Status	0.05	0.08	0.59	0.18	0.07	2.55	0.011	1.20	1.04	1.38	0.05	0.05	0.99	0.322	1.05	0.95	1.16
Migrant Status	-0.04	0.07	-0.54	-0.40	0.06	-6.38	0.000	0.67	0.59	0.76	0.02	0.04	0.44	0.661	1.02	0.94	1.11
Ever Married	0.14	0.11	1.28	0.49	0.10	4.86	0.000	1.63	1.34	1.98	0.84	0.08	11.03	0.000	2.32	2.00	2.69
Number of Sisters	0.01	0.02	0.43	0.09	0.02	4.41	0.000	1.09	1.05	1.13	0.11	0.01	9.04	0.000	1.12	1.09	1.15
Average Market Integration	0.02	0.09	0.18	-0.18	0.07	-2.67	0.008	0.83	0.73	0.95	-0.14	0.05	-2.87	0.004	0.87	0.79	0.96
Proportion of Migrants	-1.74	1.14	-1.53	-1.18	0.86	-1.38	0.168	0.31	0.06	1.65	-0.07	0.65	-0.12	0.908	0.93	0.26	3.31
**Random Effects**	**Variance**	**Std.Dev.**		**Variance**	**Std.Dev.**						**Variance**	**Std.Dev.**					
(Intercept)	0.12	0.34		0.06	0.24						0.03	0.18					
Residual	1.98	1.41		n/a	n/a						n/a	n/a					
**Model type**	**Linear**			**Binomial**							**Binomial**						
Number of observations	1972			1637							1854						
Number of groups	22			22							22						

Supplementary Table 9. Multilevel regression estimates, standard errors and (where appropriate) odds ratios and 95% confidence intervals from the models of (a) Social Network Size, (b) Density and (c) Kinship Density including the proportion of migrants as a covariate at the community level of the models.

Fixed Effects	(a) Social Network Size Estimate	Std. Error	t value
Intercept	2.80	0.16	17.82
Relative Age	-0.11	0.06	-1.83
Relative Age sq	-0.06	0.04	-1.46
Relative SES	0.12	0.05	2.33
Relative Material Wealth	0.12	0.04	2.93
Absolute Market Integration	0.02	0.04	0.42
Relative Farming Wealth	0.00	0.04	0.03
Farmer Status	0.05	0.08	0.55
Migrant Status	-0.04	0.07	-0.62
Ever Married	0.14	0.11	1.29
Number of Sisters	0.01	0.02	0.45
Average Market Integration	0.03	0.09	0.38
**Random Effects**	**Variance**	**Std.Dev.**	
(Intercept)	0.13	0.36	
Residual	1.98	1.41	
**Model type**	**Linear**		
Number of observations	1972		
Number of groups	22		

Fixed Effects	(b) Density Estimate	Std. Error	z-value	Pr(>|z|)	OR	95% CI	
Intercept	0.58	0.14	4.11	0.000	1.78	1.35	2.34
Relative Age	0.20	0.05	3.73	0.000	1.22	1.10	1.36
Relative Age sq	0.33	0.05	7.19	0.000	1.40	1.28	1.53
Relative SES	-0.05	0.05	-1.15	0.249	0.95	0.87	1.04
Relative Material Wealth	-0.07	0.04	-1.71	0.087	0.93	0.87	1.01
Absolute Market Integration	0.04	0.04	1.04	0.300	1.04	0.97	1.12
Relative Farming Wealth	0.03	0.04	0.72	0.471	1.03	0.95	1.11
Farmer Status	0.18	0.07	2.52	0.012	1.20	1.04	1.38
Migrant Status	-0.41	0.06	-6.46	0.000	0.67	0.59	0.75
Ever Married	0.49	0.10	4.83	0.000	1.62	1.33	1.98
Number of Sisters	0.09	0.02	4.41	0.000	1.09	1.05	1.13
Average Market Integration	-0.18	0.07	-2.42	0.015	0.84	0.72	0.97
**Random Effects**	**Variance**	**Std.Dev.**					
(Intercept)	0.07	0.27					
Residual	n/a	n/a					
**Model type**	**Binomial**						
Number of observations	1637						
Number of groups	22						

Fixed Effects	(c) Kinship Density Estimate	Std. Error	z-value	Pr(>|z|)	OR	95% CI	
Intercept	-1.43	0.10	-14.33	0.000	0.24	0.20	0.29
Relative Age	-0.08	0.04	-2.06	0.039	0.93	0.86	1.00
Relative Age sq	-0.01	0.03	-0.57	0.570	0.99	0.94	1.04
Relative SES	-0.11	0.03	-3.26	0.001	0.90	0.84	0.96
Relative Material Wealth	-0.02	0.03	-0.70	0.484	0.98	0.94	1.03
Absolute Market Integration	-0.12	0.03	-4.61	0.000	0.89	0.85	0.93
Relative Farming Wealth	-0.02	0.02	-0.88	0.379	0.98	0.94	1.03
Farmer Status	0.05	0.05	1.00	0.318	1.05	0.95	1.16
Migrant Status	0.02	0.04	0.44	0.660	1.02	0.94	1.11
Ever Married	0.84	0.08	11.08	0.000	2.33	2.00	2.70
Number of Sisters	0.11	0.01	9.05	0.000	1.12	1.09	1.15
Average Market Integration	-0.09	0.05	-1.88	0.060	0.91	0.83	1.00
**Random Effects**	**Variance**	**Std.Dev.**					
(Intercept)	0.03	0.18					
Residual	n/a	n/a					
**Model type**	**Binomial**						
Number of observations	1854						
Number of groups	22						

Supplementary Table 10. Multilevel regression estimates, standard errors and (where appropriate) odds ratios and 95% confidence intervals from the models of (a) Social Network Size, (b) Density and (c) Kinship Density using uncentered versions of the market integration variable at the household level.
