# Supplementary material for: Market integration reduces kin density in women’s ego-networks in rural Poland
Source: Nat Commun. 2020 Jan 14;11:266. doi: 10.1038/s41467-019-14158-2 (PMC6959218; doi:10.1038/s41467-019-14158-2)
Supplement: Supplementary file 3 — Reporting Summary [file 41467_2019_14158_MOESM3_ESM.pdf]

nature research

Corresponding author(s): Heidi Colleran

Last updated by author(s): 2019/12/14

Reporting Summary

Nature Research wishes to improve the reproducibility of the work that we publish. This form provides structure for consistency and transparency in reporting. For further information on Nature Research policies, see Authors & Referees and the Editorial Policy Checklist .

Statistics

For all statistical analyses, confirm that the following items are present in the figure legend, table legend, main text, or Methods section.

n/a	Confirmed	
☐	☒	The exact sample size (n) for each experimental group/condition, given as a discrete number and unit of measurement
☒	☐	A statement on whether measurements were taken from distinct samples or whether the same sample was measured repeatedly
☐	☒	The statistical test(s) used AND whether they are one- or two-sided<br>*Only common tests should be described solely by name; describe more complex techniques in the Methods section.*
☐	☒	A description of all covariates tested
☒	☐	A description of any assumptions or corrections, such as tests of normality and adjustment for multiple comparisons
☐	☒	A full description of the statistical parameters including central tendency (e.g. means) or other basic estimates (e.g. regression coefficient) AND variation (e.g. standard deviation) or associated estimates of uncertainty (e.g. confidence intervals)
☐	☒	For null hypothesis testing, the test statistic (e.g. F, t, r) with confidence intervals, effect sizes, degrees of freedom and P value noted<br>*Give P values as exact values whenever suitable.*
☒	☐	For Bayesian analysis, information on the choice of priors and Markov chain Monte Carlo settings
☐	☒	For hierarchical and complex designs, identification of the appropriate level for tests and full reporting of outcomes
☐	☒	Estimates of effect sizes (e.g. Cohen's d, Pearson's r), indicating how they were calculated

Our web collection on statistics for biologists contains articles on many of the points above.

Software and code

Policy information about availability of computer code

Data collection	No software was used for data collection
Data analysis	Microsoft Excel, R v 3.6 (and packages as cited in the SM)

For manuscripts utilizing custom algorithms or software that are central to the research but not yet described in published literature, software must be made available to editors/reviewers. We strongly encourage code deposition in a community repository (e.g. GitHub). See the Nature Research guidelines for submitting code & software for further information.

Data

Policy information about availability of data

All manuscripts must include a data availability statement. This statement should provide the following information, where applicable:

- Accession codes, unique identifiers, or web links for publicly available datasets
- A list of figures that have associated raw data
- A description of any restrictions on data availability

The data that support the results of this study are available on reasonable request from the author. For the protection of participant privacy I cannot make the data openly publicly available.

Field-specific reporting

Please select the one below that is the best fit for your research. If you are not sure, read the appropriate sections before making your selection.

☐ Life sciences ☒ Behavioural & social sciences ☐ Ecological, evolutionary & environmental sciences

Behavioural & social sciences study design

All studies must disclose on these points even when the disclosure is negative.

Study description	Data used for this study were collected as part of an anthropological-demographic study carried out by the author between 2009 and 2010, and which involved living in the study communities while collecting both quantitative and qualitative data. A number of Polish research assistants helped with survey data collection.
Research sample	The participants were villagers - all women - from 22 communities in rural Poland, aged between 18 and 91 (mean 44 yrs, s.d. 17.8). The study communities are situated in the valleys of the Beskid Wyspowy (Island) mountain range in the outer Western Carpathians in the Southwest of the country. The sample should not be considered representative of the country as a whole, but only representative of the wider rural population of this particular region.
Sampling strategy	The area was chosen as a study site because of its interesting demographic (e.g. high fertility) and cultural (e.g. traditional farming) features. It is one of very few regions in Europe where subsistence farming is still practiced. A power calculation performed prior to data collection indicated that 15 communities and a total sample of 1,800 individuals would be needed for multilevel analysis. This was based on the following values; a desired power value of >0.8 (i.e. the probability that the ensuing analyses can detect an effect), a conservative effect size (β) of 0.1 (the minimum level above which a statistical test can detect an effect between 2 or more groups or avoid type II errors) and an α error probability of 0.05 (the likelihood of an effect happening by chance/the probability of finding a significant relationship where none exists, i.e. avoiding type 1 errors). I used a conservative a-priori effect size as well as a 1:3 ratio of α to β (weighting type 1 errors three times more important than type II errors), to estimate the required sample size for a total of 15 groups. I opted to over-sample 22 communities based on the assumption that some would refuse to participate. The sample of communities (21 villages and one town) was randomly drawn from four neighbouring municipalities containing a total of 34 potential study populations using a random number generator in Excel. There were no available lists of inhabitants or high-resolution maps to sample from, so within each community every third house was sampled, with every adult woman (≥18 years) present in the house invited to take part in the survey. This strategy was further stratified in the town, by randomly selecting streets from a list obtained from the local government and approaching every third house/apartment on that particular street. We returned to houses that were unoccupied at the time of selection on up to three occasions. All consenting adult women were interviewed. Non-consenting or ineligible women were counted as non-responders. This sampling strategy means that important variables such as age are normally distributed in all groups. The within-community samples are representative of the villages women inhabit and the aggregates used (community-level variables) in the analyses are therefore appropriate to the research question.
Data collection	Pen and paper questionnaires were used to collect the quantitative data that support this study. Survey interviews were semi-structured, conducted in Polish in the home of the participant or a place of her choosing, and were typically conducted with a second interviewer present to provide quality control. A team of research assistants helped to collect these survey data. Research assistants were not aware of the specific research hypotheses.
Timing	Data were collected between June to September 2009 and March to November 2010.
Data exclusions	As described in the main text, 23 women who were not living in the 22 study communities were excluded from the statistical analyses, reducing the total sample size from 1995 to 1972 individuals.
Non-participation	The response rate at the individual level was 75% (ranging from 52% to 89% depending on the community). The response rate at the community level was 100%.
Randomization	Participants were not allocated into randomised or experimental groups.

Reporting for specific materials, systems and methods

We require information from authors about some types of materials, experimental systems and methods used in many studies. Here, indicate whether each material, system or method listed is relevant to your study. If you are not sure if a list item applies to your research, read the appropriate section before selecting a response.

Materials & experimental systems

n/a	Involved in the study	
☒	☐	Antibodies
☒	☐	Eukaryotic cell lines
☒	☐	Palaeontology
☒	☐	Animals and other organisms
☐	☒	Human research participants
☒	☐	Clinical data

Methods

n/a	Involved in the study	
☒	☐	ChIP-seq
☒	☐	Flow cytometry
☒	☐	MRI-based neuroimaging

nature research | reporting summary

October 2018

Human research participants

Policy information about studies involving human research participants

Population characteristics	See above.
Recruitment	Once a house was selected for participation, individual participants were recruited at home.
Ethics oversight	Ethics oversight and clearance was provided by the Department of Anthropology, University College London, UK.

Note that full information on the approval of the study protocol must also be provided in the manuscript.
